# High-throughput assessment of context-dependent effects of chromatin proteins

**DOI:** 10.1186/s13072-016-0096-y

**Published:** 2016-10-18

**Authors:** Laura Brueckner, Joris van Arensbergen, Waseem Akhtar, Ludo Pagie, Bas van Steensel

**Affiliations:** 1 Division of Gene Regulation, Netherlands Cancer Institute, Amsterdam, The Netherlands; 2 Division of Molecular Genetics, Netherlands Cancer Institute, Amsterdam, The Netherlands; 3 Department of Cell Biology, Erasmus University Medical Center, Rotterdam, The Netherlands

**Keywords:** HP1a, *Drosophila melanogaster*, Transcriptional repression, Chromatin states, Epigenetic memory

## Abstract

**Background:**

Chromatin proteins control gene activity in a concerted manner. We developed a high-throughput assay to study the effects of the local chromatin environment on the regulatory activity of a protein of interest. The assay combines a previously reported multiplexing strategy based on barcoded randomly integrated reporters with Gal4-mediated tethering. We applied the assay to *Drosophila* heterochromatin protein 1a (HP1a), which is mostly known as a repressive protein but has also been linked to transcriptional activation.

**Results:**

Recruitment to over 1000 genomic locations revealed that HP1a is a potent repressor able to silence even highly expressing reporter genes. However, the local chromatin context can modulate HP1a function. In pericentromeric regions, HP1a-induced repression was enhanced by twofold. In regions marked by a H3K36me3-rich chromatin signature, HP1a-dependent silencing was significantly decreased. We found no evidence for an activating function of HP1a in our experimental system. Furthermore, we did not observe stable transmission of repression over mitotic divisions after loss of targeted HP1a.

**Conclusions:**

The multiplexed tethered reporter assay should be applicable to a large number of chromatin proteins and will be a useful tool to dissect combinatorial regulatory interactions in chromatin.

**Electronic supplementary material:**

The online version of this article (doi:10.1186/s13072-016-0096-y) contains supplementary material, which is available to authorized users.

## Background

Eukaryotic genomes are packaged in various types of chromatin that each has specific roles in the regulation of gene expression and other nuclear functions. These chromatin types (or “states”) are defined by their distinct but sometimes partially overlapping protein compositions [1, 2]. One of the main challenges in chromatin biology is to understand the combinatorial logic of chromatin proteins: How does the regulatory function of each protein depend on the presence or absence of other proteins?

This question is exemplified by HP1, a key component of classical heterochromatin. HP1 binds to di- or tri-methylated lysine 9 of histone H3 (H3K9me2/3) and can package nucleosomal DNA into a conformation that is able to repress transcription [3–5]. Several observations indicate that the regulatory activity of HP1 is context-dependent. In *Drosophila*, the archetype ortholog HP1a localizes not only to pericentromeric regions where it is thought to contribute to the silencing of transposable elements, but also to a subset of transcriptionally active genes scattered along the chromosome arms [6, 7]. Certain genes even appear to be activated by HP1a [8, 9]. How this context-dependency arises is largely unknown, but many proteins have been identified that either promote or counteract heterochromatin formation [10]. An example is JIL-1 kinase, which is able to phosphorylate serine 10 of histone H3, a modification that blocks the interaction of HP1 with H3K9me2/3 [11, 12].

Here, we present a method to systematically study how the regulatory activity of a protein may depend on the local chromatin context. The method combines barcoded, randomly integrated reporter genes [13] with artificial tethering of a protein of interest [14–16] to these reporter genes. More than 1000 random integration sites throughout the genome offer the required statistical power to infer how various chromatin environments may influence the regulatory activity of the protein of interest. We illustrate this approach using *Drosophila* HP1a as a model.

## Results

### Experimental design

**Fig. 1:**
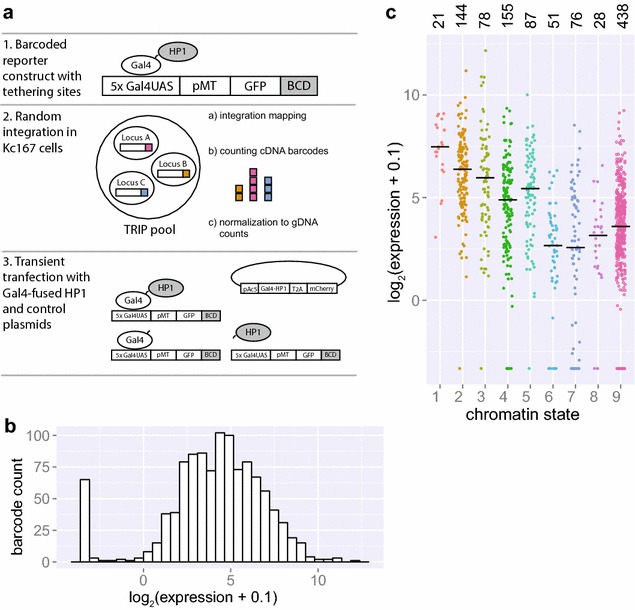
Chromatin effects on gene expression assessed by thousands of reporters integrated in parallel. **a** Principle of thousands of reporters integrated in parallel (TRIP) coupled with targeted recruitment of Gal4-fused proteins. **b** Distribution of reporter expression in the Gal4-transfected control condition as quantified by NGS. **c** Expression of integrated reporters as quantified by NGS in the Gal4-transfected control condition divided over nine chromatin states. Median values are represented by *black horizontal bars*. Number of reporters integrated in each state is specified above graph. Data information: In (**b**–**c**), a pseudocount of 0.1 was added to all expression values in order to be able to visualize non-expressed reporters on a log scale

Our approach builds on the previously reported TRIP protocol [13], which begins with transposase-mediated random genomic integration of reporter constructs in a pool of cells. All reporters are identical except for a short random barcode sequence within the transcription unit. In the resulting pool of cells, each reporter integration is mapped by a next-generation sequencing (NGS) approach to its genomic location and linked to its unique barcode sequence. Finally, NGS-based counting of barcodes in mRNA isolated from the cell pool enables us to determine the relative expression level of each reporter. By combining this information with the location of each reporter, we can study chromatin position effects in high throughput. As an addition to this original TRIP protocol, we inserted five copies of the Gal4UAS motif upstream of the promoter of our integrated reporters. This makes it possible to tether a fusion protein consisting of GalDBD and a chromatin protein of interest, here HP1a. (Fig. 1a). For brevity, Gal4DBD and HP1a will be referred to as Gal4 or HP1. Various studies in *Drosophila* have previously shown that tethered HP1 can cause silencing of a reporter gene [17–20].

We used a reporter construct consisting of a green fluorescent protein (GFP) under the control of the copper-inducible metallothionein promoter (pMT). We randomly integrated this construct via Sleeping Beauty transposition in *Drosophila* Kc167 cells. We chose this particular cell line because extensive maps of histone marks, chromatin protein binding and computationally defined chromatin states are available. In the resulting TRIP cell pool, we were able to map 1093 integrations and link them to unique barcodes. We then induced the pMT by adding 0.5 mM CuSO_4_ to the TRIP cell pool. Two days after induction, we transiently transfected the pool with a plasmid expressing Gal4-HP1, or unfused Gal4 or HP1 as controls. These vectors also express mCherry, which enabled us to assess transfection levels and to isolate transfected cells by fluorescence-activated cell sorting (FACS). Two days after transfection, we collected transfected cells and extracted mRNA and genomic DNA (gDNA) for barcode counting by NGS. In order to compare expression between samples and to determine absolute up- or downregulation, we used a spike-in consisting of a low-complexity independent TRIP cell library. After normalization (see “Methods”), we observed good correlations between two independent transfection experiments (Additional file 1: Figure S1). For downstream analyses, we averaged the normalized expression values of these two replicates for each reporter integration.

### Integrated reporters reflect the local chromatin state

First, we examined position effects on the expression of the integrated TRIP reporters in the absence of tethered HP1. For this, we used the Gal4-only transfected control cell pools. This revealed an approximately 1000-fold variation in reporter expression (Fig. 1b), similar to what was previously observed by TRIP in mouse cells [13]. This demonstrates strong position-dependence of reporter expression.

To investigate whether this variation in expression could be explained by differences in the local chromatin environment, we overlaid the TRIP data with a chromatin state map in which chromatin was subdivided into nine states according to combinatorial patterns of histone modifications [2]. Briefly, states 1–5 represent various chromatin states associated with active transcription; state 6 is enriched in the polycomb-associated mark H3K27me3; state 7 corresponds primarily to pericentromeric heterochromatin and is highly enriched in HP1a, H3K9me2/me3 and the corresponding histone methyltransferase Su(var)3-9; state 8 describes heterochromatin-like regions present on autosomal arms and with lower HP1 and Su(var)3–9 occupation than state 7; and finally, state 9 covers 40 % of the genome and is mostly devoid of histone marks [2].

We observed more than 30-fold differences in the average expression levels of reporters across the nine chromatin types (Fig. 1c). Mean reporter expression was highest in states 1 and 2 which describe transcriptionally active non-intronic regions. Expression was lowest in polycomb-marked state 6 and heterochromatin states 7 and 8. These observations are in accordance with the previously described association of these chromatin types with active or inactive transcription [2] and indicate that integrated reporters are strongly influenced by the state of the surrounding chromatin. We obtained similar results with an alternative five-state chromatin model [1], which showed up to tenfold differences in mean reporter expression depending on the chromatin state (Additional file 2: Figure S2).

### Tethering HP1 results in global downregulation of integrated reporters

Next, we determined the global effect of tethering of HP1 to the integrated reporters. We first measured GFP expression by flow cytometry on days 2, 4 and 6 after transfection with Gal4-HP1, Gal4 or HP1. GFP levels were visibly reduced by day 4 after Gal4-HP1 transfection compared to both controls and stayed low until day 6 (Additional file 3: Figure S3). We decided to focus on day two after transfection, because at later time points the HP1-induced repression might have become too strong to observe position-dependent differences. We measured bulk GFP expression at the mRNA level across all reporters in the cell pool by conventional RT-qPCR analysis. This yielded ratios of 0.33 for Gal4-HP1/Gal4, 0.30 for Gal4-HP1/HP1 and 0.92 for HP1/Gal4. Thus, tethering of HP1 results in an overall downregulation of reporter RNA levels, whereas expression of untethered HP1 does not have a major effect.

**Fig. 2:**
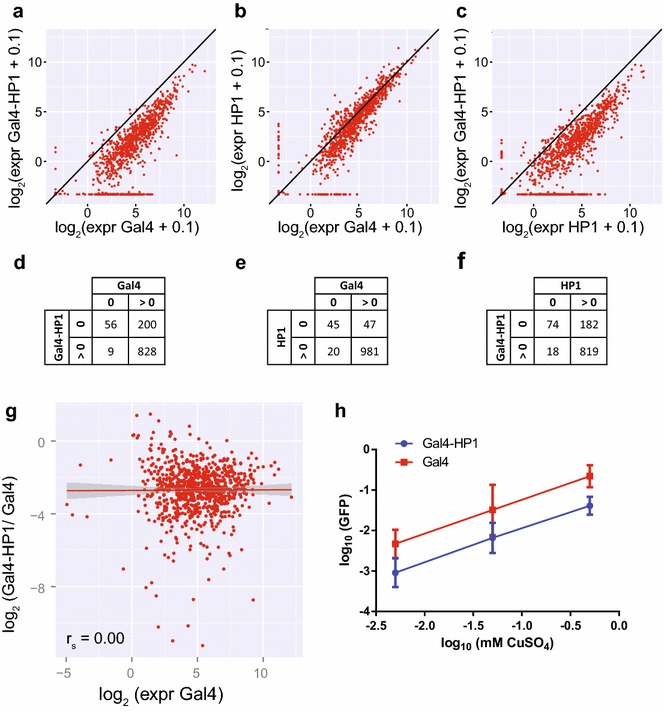
Tethering of Gal4-HP1 results in global downregulation of integrated reporters. **a**–**c** Reporter expression compared between TRIP pool transfected with Gal4-HP1 or controls Gal4 or HP1 only as quantified by NGS 2 days after transfection. **d**–**f** Quantification of reporters showing expression detectable by at least one sequencing read. **g** Correlation (Spearman) between expression in the Gal4-transfected control condition and downregulation upon HP1 tethering with linear regression (*red*) and standard error. Downregulation is quantified by ratio of expression in the Gal4-HP1-transfected condition over expression in the Gal4-transfected control condition; therefore, only reporters with detectable expression in both conditions are shown. **h** GFP reporter expression in a cell line with single integration induced with increasing levels of CuSO_4_ and transfected with Gal4-HP1 or Gal4 as quantified by qPCR. Amount of repression upon Gal4-HP1 tethering is constant over expression range. Data information: In (**a**–**c**), a pseudocount of 0.1 was added to all expression values in order to be able to visualize non-expressed reporters on a log scale

We then investigated the effect of tethered HP1 on each individual reporter by comparing the normalized barcode expression levels in Gal4-HP1 expressing cells to those of Gal4 control cells. This revealed that most reporters are downregulated upon HP1 tethering (Fig. 2a, d). Seventy percent of reporters exhibited a greater than twofold reduction in expression, and 18 % of reporters with detectable expression in the control condition were completely silenced. For reporters with detectable expression in both conditions, the average fold change Gal4-HP1/Gal4 was 0.23 ± 0.25 (mean ± standard deviation). Transfection with HP1 alone may have a mild effect on a subset of reporters (mean fold change HP1/Gal4 0.90 ± 0.98) (Fig. 2b, e), but the repressive effect of Gal4-HP1 was consistently much stronger (mean fold change Gal4-HP1/HP1 0.35 ± 0.54) (Fig. 2c, f), which indicates that transfection with untethered HP1 does not have a major effect on bulk reporter expression. Western blot analysis indicated that the strong decrease in expression with Gal4-HP1 was not due to higher expression of the fusion protein compared to HP1 only (Additional file 4: Figure S4). This analysis also indicates that expression of endogenous HP1 was not affected by transfection with Gal4-HP1 or HP1.

Only 5.1 % of reporters were unaffected by HP1 tethering, as defined by a less than twofold change in expression. Finally, 0.27 % of all reporters were upregulated more than twofold upon HP1 tethering. Such rare events could be due to technical noise rather than a biological effect. We conclude that HP1 represses transcription when tethered upstream of a promoter, in the vast majority of genomic locations.

### Gal4-HP1-induced silencing is not correlated with transcription levels in the absence of tethering

Because we transfected the TRIP cell pool 2 days after induction of reporter expression, HP1 has to compete with the transcription machinery in order to establish a heterochromatin state. We therefore wondered whether the degree of repression was related to the initial expression level of the reporter gene. We used the Gal4-transfected control cells to estimate this initial expression (leaving out reporters without any detectable initial expression) and compared it to the extent of silencing observed with Gal4-HP1 (Fig. 2g). Strikingly, there was no correlation between expression levels of reporters and the extent of downregulation observed with Gal4-HP1 (Spearman’s *ρ* = 0.00).

We took a complementary approach to confirm that repression by HP1 is independent of the initial transcriptional activity of a gene. We generated cells with a pMT-driven GFP reporter that was integrated in a single euchromatic locus. We then induced transcription from this promoter by adding various concentrations of CuSO_4_ and determined reporter expression levels by RT-qPCR. Compared to bulk expression of TRIP reporters, we obtained 6.2-fold lower expression with the lowest level of induction and 7.5-fold higher expression with the highest CuSO_4_ concentration. Thus, we could test the effect of HP1 on a single gene over a broad range of expression levels. We then tethered Gal4-HP1 and observed a consistent fold reduction in expression of 0.22 ± 0.12, 0.28 ± 0.24 and 0.21 ± 12 from lowest to highest induction level (Fig. 2h). In conclusion, Gal4-HP1-induced silencing is equally efficient over a wide range of reporter expression levels.

### Gal4-HP1-induced silencing is increased in pericentromeric heterochromatin

**Fig. 3:**
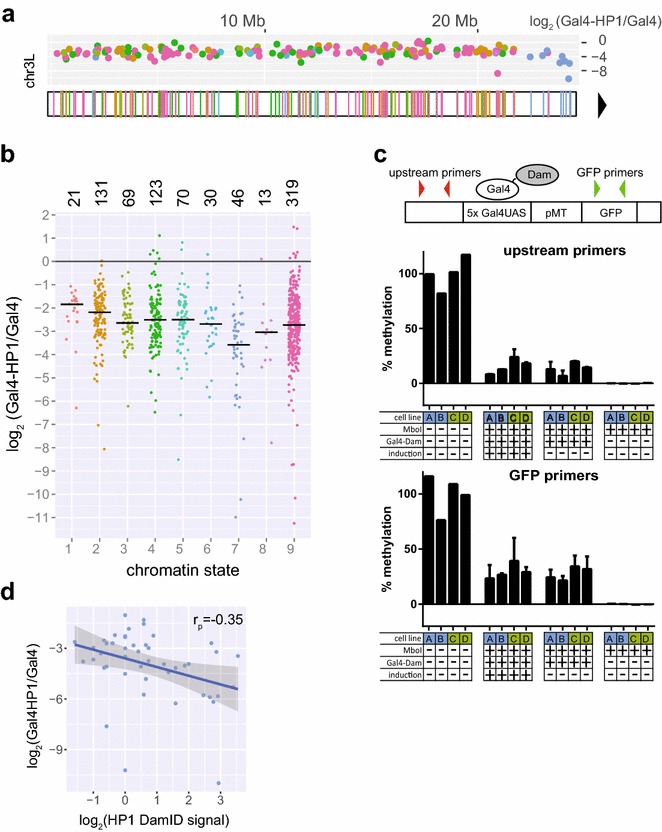
Repression upon HP1 tethering is modulated by the local chromatin environment. **a** Positions of the integration sites of TRIP reporters on chromosome 3L. *Colors* represent chromatin state at the integration site according to the nine-state model (as labeled in Fig. 3b). Centromere position is indicated by black triangle. Plot above the chromosome shows fold downregulation upon Gal4-HP1 tethering. All chromosomes are shown in Additional file 4: Figure S4. **b** Fold downregulation upon Gal4-HP1 tethering segregated by chromatin state. Median values are indicated by *black horizontal bars*. Number of reporters integrated in each state is specified above graph. **c** Quantification of reporter accessibility via tethering of Gal4-Dam in four cell lines each with a single integration. Dam-mediated adenine methylation was assessed by MboI digestion of unmethylated GATC sequences followed by qPCR. Position of qPCR primer pairs is indicated on schematic representation of reporter. *Bar graphs* show methylation levels measured in two cell lines (labeled *A*, *B*) with reporter integration in state 7 heterochromatin (*blue*) and two cell lines (labeled *C*, *D*) with integration in state 3 euchromatin (*green*). *Error bars* represent standard deviation of two independent transfections. Measurements were taken with or without reporter induction. **d** HP1 occupancy levels at reporter integrations sites in state 7 domains as quantified by DamID correlate with downregulation upon Gal4-HP1 recruitment (Pearson). Linear regression (*blue*) with standard error

Next, we were curious whether the extent of silencing upon HP1 tethering is linked to different chromatin environments. We therefore analyzed the Gal4-HP1/Gal4 fold change in reporter expression as a function of the chromatin types according to the nine-state model (Fig. 3a, b; Additional file 5: Figure S5). This revealed up to 3.5-fold variation between chromatin states, with the least repression occurring in states 1 and 2 and the strongest repression in state 7. This suggests that the local chromatin environment can modulate the ability of tethered HP1 to repress transcription.

State 7 is of particular interest because it coincides with pericentromeric regions that are densely occupied by endogenous HP1 and Su(var)3–9. Reporters in state 7 exhibited a significantly stronger repression by tethered HP1 than reporters in the other chromatin states (2.0-fold difference in median repression levels; Wilcoxon’s test *p* = 2.3 × 10^−7^). As shown above, transcriptional activity in general did not correlate with the extent of downregulation observed upon HP1 recruitment, but since there are large differences in reporter expression between chromatin states we wanted to exclude it as possible explanation for the observed difference in silencing. We therefore picked reporters from other chromatin types with expression levels most closely matching those of the reporters in state 7 and compared the Gal4-HP1/Gal4 fold change in expression (we excluded four reporters in state 7 that were outside of the expression range of the remaining reporters and could therefore not be matched). Again, we observed a significantly stronger repression for reporters in state 7 (2.2-fold difference in median repression levels, Wilcoxon’s test *p* = 2.8 × 10^−5^). Repression by Gal4-HP1 was also significantly stronger in state 7 than in other states when normalized to untethered HP1 (Wilcoxon’s test *p* = 0.0077, Additional file 6: Figure S6). We conclude that state 7 chromatin provides a favorable environment for repression by tethered HP1, irrespective of the initial activity of the reporter.

Although heterochromatin is generally thought to be less accessible, we checked whether the targeting of Gal4 fusion proteins was somehow more efficient in state 7 chromatin. We tested this by DamID of Gal4 using a previously reported qPCR-based readout [21]. We performed this assay in two cell lines with a single reporter integration in state 7 heterochromatin and two cell lines with integrations in state 3 euchromatin (Fig. 3c). As expected, state 7 integrations did not show higher accessibility for Gal4 binding as measured by methylation levels.

To explain the enhanced downregulation in state 7, we examined the occupancy of endogenous HP1 at reporter integrations sites by DamID profiles. HP1 is markedly enriched in state 7 pericentromeric heterochromatin, with fourfold higher levels compared to all other states combined. Within state 7, the fold downregulation upon tethering correlates with local HP1 binding levels (Pearson’s *r* = −0.35, *p* = 0.018) (Fig. 3d) but not in other states. We also confirmed by immunofluorescence microscopy that Gal4-HP1 as well as control HP1 accumulated in the chromocenter that contains high concentrations of endogenous HP1 (Additional file 7: Figure S7). This homing behavior in combination with high availability of endogenous HP1 in pericentric heterochromatin might facilitate the repressive action.

### Gal4-HP1-induced silencing is less effective in chromatin associated with elongating transcription

Silencing by tethered HP1 was least effective in chromatin states 1 and 2, with median repression reduced by 1.8- or 1.4-fold, respectively, compared to reporters in other chromatin states. State 2 gave a significant test result versus other chromatin states (Wilcoxon’s test *p* = 0.00022) and also when comparing reporters of matching expression (*p* = 0.0094). State 1 resulted in a *p* value of 0.016 when comparing downregulation with all other chromatin types but did not give a significant result in the expression-matched test (*p* = 0.48). We therefore focused on state 2. This state is typically present on exonic regions of transcribed genes and associated with high levels of H3K36me3 and H3K79me2 [2]. Indeed, we observed significantly reduced repression of reporters integrated in H3K36me3-bound regions (1.4-fold difference in median repression, *p* = 0.00028, *N* = 118). H3K36me3 also significantly correlated with fold change Gal4-HP1/Gal4 when using continuous ChIP-seq data (*p* = 2.3 × 10^−5^, Spearman’s *ρ* = 0.15). We obtained similar results for H3K79me2 (1.3-fold difference in median repression, *p* = 0.00060, *N* = 135). It will be interesting to investigate whether these histone marks are directly responsible for the inhibitory effect on HP1-induced silencing, or mere indirect correlates. Finally, we also found a significant correlation with ChIP scores for JIL-1, a H3S10 kinase known to limit heterochromatin spreading (*p* = 0.0014, Spearman’s *r ρ* = 0.11) [22].

### Variegation upon HP1 tethering

**Fig. 4:**
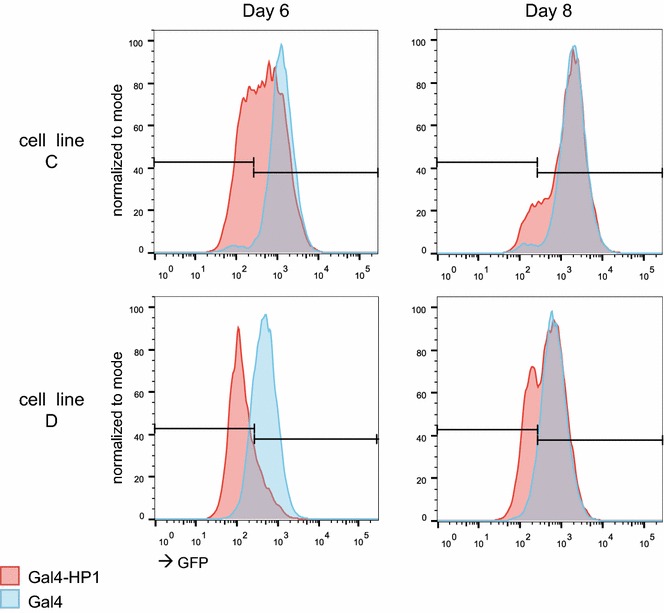
Variegation upon HP1 targeting. GFP expression distributions in two single-integration cell lines upon Gal4-HP1 targeting (*red*) compared to Gal4 only (*blue*) as quantified via FACS by 6 and 8 days after transient transfection

HP1-containing heterochromatin is known for its ability to cause variegating patterns of gene repression, with the target gene being either “on” or “off” [23]. The degree of repression of a particular TRIP reporter may therefore reflect the proportion of cells in the “off” state, rather than downregulation along a continuous scale. Because TRIP cannot discriminate between these two different modes of repression, we tested the effect of Gal-HP1 tethering by FACS analysis of two cell lines with stably integrated single-copy pMT-driven GFP reporters (Fig. 4). As in the TRIP experiments, we first activated the pMT promoter and then transfected the cells with Gal-HP1 or control plasmids. After 6–8 days, both lines showed bimodal GFP expression distributions after Gal-HP1 tethering, with the highest expression mode roughly coinciding with a single peak of expression observed in control cells that were transfected with Gal4 alone. However, the two cell lines differed in the proportion of cells in the “off” state, and this proportion was also dependent on the time point at which the cells were analyzed. This result indicates that tethered HP1 causes variegating expression, with the balance between “on” and “off” depending on the local chromatin environment. Considering the strong similarity of the reporter construct used for this experiment and the one used in the TRIP studies, it is likely that variegating expression also occurs in the case of TRIP reporters after Gal-HP1 tethering.

### Gal4-HP1-induced silencing does not result in permanent memory

**Fig. 5:**
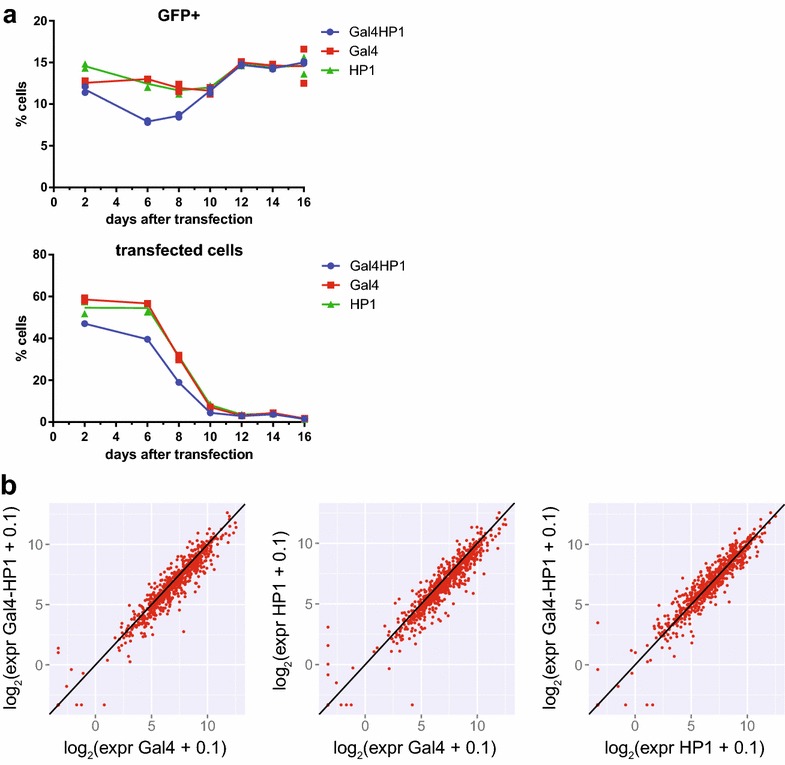
Tethering of HP1 does not result in permanent repression. **a** GFP expression of TRIP pool and amount of transfected cells over time after transient transfection with Gal4-HP1 or control plasmids as quantified by FACS. Graphs show data from two replicate experiments with line connecting means. **b** Reporter expression compared between TRIP pool transfected with Gal4-HP1 or controls Gal4 or HP1 only as quantified by NGS 16 days after transfection

Targeting of exogenous HP1 can induce a mitotically heritable heterochromatin state in mammalian cells [24, 25]. We were therefore curious whether we could observe heritability of Gal4-HP1-induced silencing after loss of tethered HP1 in the around 1000 reporters in our *Drosophila* cell system. To test this, we kept the TRIP cell pool transiently transfected with Gal4-HP1 and control plasmids in culture until the cells had lost plasmid expression. Initial transfection efficiencies, as quantified by FACS, were approximately 50 % for all plasmids (Fig. 5a). By day 6, we still measured 40–55 % transfected cells. At this time point, in the Gal4-HP1-transfected replicates 7.8 and 8.0 % of the cells were GFP positive, compared to 12–13 % in the Gal4- and HP1-transfected control samples. This confirmed that tethered HP1 induced silencing of GFP expression. By day 16, we measured 1.3–2.1 % remaining mCherry-positive cells, indicating that the TRIP cell pool had mostly lost the transfected plasmids. At this time point, 15 % of cells in the duplicate cultures that were initially transfected with Gal4-HP1 were GFP positive, which is comparable to 13–17 % GFP-positive cells in the control samples. This indicated that most integrated reporters had recovered from Gal4-HP1-induced silencing. We collected these cell pools and quantified individual barcode expression in mRNA by NGS (Fig. 5b). The average fold expression change for all barcodes was 0.92 ± 0.53 for Gal4-HP1/Gal4 (mean ± standard deviation), 1.2 ± 0.72 for Gal4-HP1/HP1 and 0.89 ± 0.55 for HP1/Gal4. Moreover, statistical analysis using *limma* [26] did not detect any individual reporters with significant repression after 16 days, nor did we observe significant differences in Gal4-HP1/Gal4 ratios (Wilcoxon’s test) when the data were aggregated by the nine chromatin states. We therefore conclude that there is no stable mitotic transmission of Gal4-HP1-induced silencing by 16 days after transient transfection.

## Discussion

### HP1 as a potent and universal transcriptional repressor

Our results indicate that HP1a is a potent transcriptional repressor that can reduce the activity of integrated reporters in most genomic contexts. In previous studies employing targeted recruitment of HP1 to integrated reporters in *Drosophila*, tethering did not result in silencing in a number of cases. In one study, three Gal4 binding sites were inserted between the *white* and *lacZ* reporter genes and targeted by Gal4-HP1 expressed under heat-shock promoter HSHP1-83C. In this setup, the white reporter was silenced at only one of six genomic locations tested and the one site that supported silencing was adjacent to repetitive sequences [20]. In another study, LacI-HP1 was expressed under the Hsp70 promoter and tethered to 256 lac repeats which resulted in silencing of the white reporter gene in 25 out of 26 cases as observed by white eye color [17]. In our system, Gal4-HP1 is expressed under the control of the Actin5c promoter and tethered to five repeats of the Gal4UAS motif. We did not identify a genomic environment that completely inhibited HP1. Use of a strong constitutive promoter for Gal4-HP1 expression could explain why we observe this highly efficient silencing.

Several reports have linked HP1 in *Drosophila* with an activating effect on transcription [8, 27–29]. In our study, we observed upregulation greater than twofold in only three of 1093 reporters which did not allow us to draw general conclusions. It is possible that HP1 can activate transcription in an indirect fashion or that this effect depends on HP1 localization within gene bodies, which we cannot test in our current system.

### Silencing by tethered HP1 does not depend on transcription level of the target gene

Surprisingly, the magnitude of the repression by HP1 (fold change in reporter activity) is unaffected by even high levels of transcription of the reporter. This argues against a competition model in which the transcriptional machinery, if active enough, can overrule heterochromatin. Rather, HP1a turns down transcription by a nearly constant factor, irrespective of the initial transcription levels. Although the TRIP readout is not suitable to detect cell-to-cell variation in expression, our analysis of single integrated GFP reporters indicates that HP1a causes variegation. Reduced expression as detected by TRIP should therefore be considered to reflect that the reporters are turned “off” in a proportion of the cells. This proportion is then largely independent of the initial transcription activity of the reporter.

### Chromatin environments modulate HP1 action

While the activity of the reporter itself did not affect HP1 action, we did observe quantitative differences in HP1 effects between chromatin states. Repression by HP1 was slightly but significantly less efficient in chromatin state 2, which is an exon-biased chromatin environment associated with transcriptional elongation and high levels of H3K36me3. Because H3K36 methylation is deposited co-transcriptionally [30, 31], it is difficult to determine by correlative studies whether the histone mark, RNA Pol II or other factors inhibit HP1 action. One candidate is cyclin-dependent kinase 12 (CDK12), a transcription elongation-associated RNA Pol II kinase that was recently reported to antagonize heterochromatin in *Drosophila* [32]. We also found reduced silencing in regions bound by the H3S10 kinase JIL-1, in agreement with earlier observations [22].

Our data indicate that pericentric heterochromatin facilitates repression by tethered HP1. Cooperative interactions with endogenous HP1 or its partner proteins are likely to account for this effect, because the repression by Gal-HP1 correlates with heterochromatin domain size and local density of endogenous HP1. Notably, in this study we only covered uniquely mappable reporters. It might be interesting to examine integrations in repetitive sequences.

### Absence of epigenetic memory after transient HP1 recruitment

Induction of a mitotically stable heterochromatin state by tethering of HP1 has been observed in mammalian cells [24, 25]. In fission yeast, transient overexpression of Swi6, the yeast homolog of HP1 resulted in mitotically and meiotically stable silencing at the mating-type locus [33]. H3K9 methylation nucleated at this locus could be heritably maintained after loss of the nucleation center but only in the presence of Swi6 [34]. In other studies in fission yeast, a silenced chromatin state triggered by Gal4-mediated targeting of H3K9 methylation could be maintained over mitotic and meiotic divisions. Heritability required deletion of the corresponding demethylase and the presence of Swi6 [35, 36]. In our experiments, HP1 targeting did not result in heritable silencing in any chromatin environment, as reporters ultimately regained expression levels equivalent to the unsilenced control. In murine embryonic stem cells, induction of high levels of DNA methylation was necessary for maintaining transcriptional silencing after loss of HP1 targeting and required 4 weeks of continuous HP1 recruitment. In the absence of stable tethering, HP1 was ultimately overcome by transcription [24]. We introduced HP1 via transient transfection which resulted in tethering for approximately 1 week. The short duration of HP1 targeting and the absence of canonical DNA methyltransferases Dnmt1 or Dnmt3 might explain the lack of stable silencing in our *Drosophila* system. It will be interesting to conduct tethered TRIP experiments in mammalian cells to study the chromatin context effects on such epigenetic phenomena.

## Conclusions

### Tethering TRIP as a tool to study context-dependent effects of regulatory proteins

This study provides proof of principle for the use of TRIP in combination with protein tethering to investigate how specific regulatory proteins interact with their local environment to control gene activity. As Gal4 fusions of a protein of interest can easily be generated and transfected, a wide variety of chromatin proteins may be studied by this approach. Potential differences in transfection levels between individual cells are compensated for by FACS-based isolation of 10 × 10^6^ cells which represents a theoretical coverage of around 300× of the initial barcode complexity.

The near-random integration of Sleeping Beauty transposons [37] combined with the multiplexed barcode readout makes it possible to survey most of the commonly occurring chromatin states with sufficient statistical power. Furthermore, TRIP is compatible with many reporter designs that may be used to probe a variety of functions [13]. For example, by varying the spacing between the Gal4-binding sites and the promoter of the reporter it may be possible to test the distance over which a tethered protein can exert its regulatory effects (e.g., through spreading of a chromatin state), and how this distance may depend on the local chromatin environment.

We cannot rule out that the integrated reporter in some instances alters the local chromatin state of the integration site. However, the ~1000-fold range in expression levels of the untethered reporter indicates that the local environment strongly controls the reporter. It is thus likely that the tethered protein is exposed to the same local influences. We expect that TRIP in combination with protein tethering will be a useful tool to further explore the context-dependent functions of regulatory proteins.

## Methods

### Cell culture

Kc167 cells were cultured at 23.5 °C in Shields and Sang M3 Insect Medium (Sigma-Aldrich) with 0.25 % Bacto peptone (BD), 0.1 % yeast extract (BD), 5 % heat-inactivated FBS (Thermo Scientific) and 1 % penicillin/streptomycin (Thermo Scientific). For pMT induction, sterile-filtered CuSO_4_ (Sigma-Aldrich) dissolved in H_2_O was added to 0.5 mM final concentration unless mentioned otherwise. The original source of the Kc167 cells used in this study cannot be traced, but the cells have been used in our laboratory for about 15 years, e.g., in [1, 38–40].

### Constructs

The TRIP vector was derived from Addgene plasmid #65488 by exchanging pHsp with 5x Gal4UAS—pMT via restriction enzyme cloning with EcoRV/EcoRI. pMT was derived from a commercially available pMT/V5-His/lacZ plasmid (Thermo Scientific), and five Gal4UAS sites (CGGAGTACTGTCCTCCGAG) were added as an oligonucleotide. Additionally, a second I-CeuI cutting site was integrated upstream of the IR-DR(R) sequence by PCR using primers 160JvA and 161JvA, followed by self-ligation.

Plasmids for expressing the Gal4-HP1 fusion and controls as well as Gal4-Dam were derived from the STABLE 2 vector for bicistronic expression via the T2A peptide, a gift from Jim Sutherland [41]. The NeoR gene in the template was replaced with mCherry and the EGFP gene with Gal4DBD-V5-HP1a, Gal4DBD-V5, V5-HP1a or Gal4DBD-Myc-Dam by Gibson assembly generating pAc5-Gal4-V5-HP1a-T2A-mCherry, etc. Gal4DBD (1-147) was copied from Addgene plasmid #43969, mCherry from mCherry-G9a [42], HP1 and Myc-Dam from pDamHP1 [21]. V5 tag (GGTAAGCCTATCCCTAACCCTCTCCTCGGTCTCGATTCTACG) was added as an oligonucleotide.

The plasmid encoding Sleeping Beauty transposase is deposited as Addgene #65487.

Plasmids expressing Dam-HP1 or Dam were previously reported [21].

### Establishment of TRIP plasmid library

A 21-nt random barcode was added to the TRIP vector by PCR amplification with Phusion polymerase (2 U, Thermo Fisher) in GC buffer with 10 ng template plasmid, 500 nM forward and reverse primer (JvA168 and JvA169) and dNTPs (250 µM each) in a total volume of 100 µl. PCR conditions were 1 min at 98 °C (1×), 15 s at 98 °C, 30 s at 55 °C, 4 min at 72 °C (30×). PCR products were treated with T4 polymerase (9 U, NEB) with added dNTPs (250 µM each) for 20 min at 12 °C to complement barcode sequences. The reaction was stopped by adding EDTA to a final concentration of 10 mM and heat inactivation for 20 min at 75 °C. Products were purified using ISOLATE II PCR and Gel kit (Bioline) according to the manufacturer’s instructions and digested with DpnI (20 U, NEB) in buffer 4 for 1 h at 37 °C in a volume of 50 µl to remove the template. The reaction was terminated by heat inactivation for 20 min at 75 °C, and barcoded fragments were circularized over night at 12 °C with T4 ligase (50 U, NEB) in T4 ligase buffer in a total volume of 900 µl. After ligation, remaining unligated fragments were removed by treatment with Plasmid-Safe DNase (40 U, Epicentre) with added ATP (final concentration 1 mM) in the manufacturer-supplied reaction buffer in a total volume of 1500 µl for 5 h at 37 °C. The reaction was terminated by heat inactivation for 30 min at 70 °C. Products were purified by two times phenol/chloroform extraction followed by ethanol precipitation.

70 ng barcoded plasmids were transformed into 50 μl megacompetent cells (MegaX DH10B™ T1R Electrocomp™ Cells, Invitrogen) by electroporation (settings: 2 kV, capacitance = 25, capacitance extension = 250, pulse control = 200) and purified using a Genopure Maxi Kit (Roche) according to the manufacturer’s instructions.

### TRIP cell pool establishment and mapping of integrations

The barcoded reporters were integrated by transfecting 1 × 10^6^ Kc167 cells with 1 µg barcoded plasmid library and 1 µg plasmid encoding Sleeping Beauty transposase. Transposase expression was induced by four heat-shock treatments of 2.5 h at 37 °C distributed over 36 h. Transfected cells were expanded until Sleeping Beauty expression was lost. For the resulting TRIP pool, we determined the number of integrations to be 0.3 per cell based on qPCR on gDNA for GFP integrations compared to a cell line with a single integration. A subpool of 30,000 cells was taken from the TRIP pool and expanded to limit the library complexity to a maximum of 10,000 integrations.

To map the reporter integrations, gDNA was extracted from 20 × 10^6^ cells using ISOLATE II Genomic DNA Kit (Bioline) and digested with NlaIII (40 U, NEB) in buffer 4 supplemented with BSA in a total volume of 100 µl for 2 h at 37 °C. The reaction was terminated by heat inactivation for 20 min at 65 °C. Fragments were circularized over night at 12 °C using T4 ligase (100 U, NEB) in T4 ligase buffer in a total volume of 1600 µl. Products were precipitated with ethanol and purified with ISOLATE II PCR and Gel Kit (Bioline). Unligated fragments were removed by digestion with Plasmid-Safe DNase (20 U, Epicentre) with added ATP (final concentration 1 mM) in the manufacturer-supplied reaction buffer in a total volume of 100 µl for 5 h at 37 °C. The reaction was terminated by heat inactivation for 30 min at 70 °C and purified using ISOLATE II PCR and Gel Kit (Bioline). To eliminate any remaining unintegrated or fully integrated plasmid and linearize the template, products were digested with I-CeuI (5 U, NEB) in buffer 4 supplemented with BSA in a total volume of 60 µl for 2 h at 37 °C. The reaction was terminated by heat inactivation for 20 min at 65 °C and purified using ISOLATE II PCR and Gel Kit (Bioline). Products were amplified in triplicate reactions by inverse PCR using Phusion polymerase (2 U, Thermo Fisher) in GC buffer with 20 µl of I-CeuI-digested DNA, 500 nM forward and reverse primer (151AR and 219AR) and dNTPs (200 µM each) in a total volume of 100 µl. PCR conditions were 1 min at 98 °C (1×), 30 s at 98 °C, 30 s at 60 °C, 45 s at 72 °C (22×). Products were purified using ISOLATE II PCR and Gel Kit (Bioline). To add indices and adapters for next-generation sequencing, one-third of PCR products was amplified in a second PCR using Phusion polymerase (1 U, Thermo Fisher) in GC buffer with 500 nM forward and reverse primer (151AR and iPCR indexing primer) and dNTPs (200 µM each) in a total volume of 50 µl. PCR conditions were 1 min at 98 °C (1×), 30 s at 98 °C, 30 s at 60 °C, 45 s at 72 °C (10×). Products were purified using ISOLATE II PCR and Gel Kit (Bioline) and prepared for next-generation sequencing as described below.

### Establishment of integrase-mediated cassette exchange in Kc167 cell clones

The vector p81_JvA_DTR_IMCE to generate clonal cassette exchange Kc cell lines was constructed using standard molecular biology techniques and is available upon request. It is based on the TRIP vector backbone in which the sequence in between the Sleeping Beauty inverted repeats has been replaced by two full AttP sites which were PCR amplified from Addgene plasmid #13843, a gift from Ting Wu [43], in a “head-to-head” orientation to facilitate cassette exchange with a AttB-containing vector (see below). In between the AttP sites, a bicistronic neomycin/diphtheria toxin selection cassette was placed which is based on the Ac5-STABLE1-neo plasmid [41] and in which we replaced the GFP ORF by the ORF of the human diphtheria toxin receptor. To generate stable clones, 20 × 10^6^ Kc167 cells were electroporated with 20 µg of Sleeping Beauty expression vector (Addgene plasmid #65487) and 4 µg of p81_JvA_DTR_IMCE and cells were expanded for 3 weeks. Selection with 40 µl/ml G418 (Sigma-Aldrich) was started after the first week. Clones were then established by limited dilution, the number of integrations was analyzed by qPCR, and clones with more than one integration were excluded from further analysis. To identify the genomic locus of the cassette exchange site in each clone, an iPCR strategy was used. Briefly, 100 ng of gDNA was digested in a volume of 25 µl with 5 units of Nla III (NEB) for 30 min and heat inactivated for 20 min at 65 °C. Of this reaction, 5 µl was self-ligated in a volume of 40 µl with 1 unit of ligase (Roche) at 16 °C overnight. On 5 µl of the ligation material, 34 PCR cycles (15 s at 98 °C; 15 s at 60 °C; 30 s at 72 °C) were performed using primer JvA45 and JvA94. The PCR product was purified using ISOLATE II PCR and Gel Kit (Bioline), Sanger sequenced using primer 45JvA and mapped to the genome (Table 1).

**Table 1:** Integration sites in Kc167 cell clones

Cell line	Integration locus
A	chr2RHet: 3094936–3094937
B	chr2L: 16340822–16340823
C	chrX: 5784726–5784727
D	chr2R: 5436488–5436489

The donor cassette vector containing the AttB sites is based on the backbone of the pMT/V5-His *Drosophila* expression vector (ThermoFisher Scientific) to which we added the full AttB sites from piB-GFP (Addgene plasmid #13844), the metallothionein promoter of the pMT/V5-His *Drosophila* expression vector, 5× Gal4UAS sites and the GFP ORF of the TRIP vector.

Cassette exchange was performed by electroporation of 1 × 10^6^ cells with 1 µg of donor cassette and 1 µg of pBS130 (a kind gift from Tom Clandinin, Addgene plasmid # 26290). After 1 week, recombined cells were selected for 1 week by adding 0.5 μg/ml diphtheria toxin (Sigma-Aldrich) every 48 h. For the inducible expression experiment and variegation experiment, we wanted to exclude cells that might have escaped selection due to epigenetic silencing of the diphtheria toxin receptor gene. We therefore isolated GFP expressing cells of clones “C” and “D” by FACS 2 days after inducing GFP expression with 0.5 mM CuSO_4_.

### Transfection

Kc167 cells were transfected by electroporation of 20 × 10^6^ cells with 20 µg plasmid at 1000 μF/250 V or 1 Mio cells with 1 µg plasmid at 450 µF/86 V using a Gene Pulser II (BioRad).

### Flow cytometry analysis and sorting

Flow cytometry quantitative analysis was performed on an LSR FORTESSA (BD Biosciences) and processed with FlowJo software. Cells were gated based on forward and side scatter for single viable cells. Remaining cells were analyzed for GFP and mCherry levels with gates set according to wild-type cells. mCherry+ cells were sorted by using a Moflo Astrios (Beckman Coulter) and immediately resuspended in TRIsure (Bioline).

### RNA isolation and cDNA generation

RNA was extracted from TRIsure-resuspended samples by chloroform extraction and precipitated with isopropanol. For TRIP samples, the polyadenylated fraction was isolated using the Oligotex Kit (Qiagen) according to the manufacturer’s instructions. RNA samples were treated with RNase-free DNase I (2 U, Roche) in DNase buffer in a total volume of 20 µl for 30 min at 37 °C. The reaction was terminated by adding 1 µl of 25 mM EDTA and incubation at 70 °C for 15 min. cDNA was generated using Tetro cDNA Synthesis Kit (Bioline) according to the manufacturer’s instructions. For TRIP samples, a GFP-specific primer (AR152) was used instead of OligoDT (Table 2).

**Table 2:** Oligonucleotides

Name	Sequence (5′–3′)
GFP_f	AGGACAGCGTGATCTTCACC
GFP_r	CTTGAAGTGCATGTGGCTGT
upstream_f	CGTACTCCACCTCACCCATC
upstream_r	TTCATCGATACCGTCGACCT
TSR_f	CAAAGAAGCAAAAGCTGTTCCTTA
TSR_r	GCTGGAGTACAACATCTTCTTCTTGAC
160JvA	CTAAGGTAGCGAAGGCAATGCTACCAAATAC
161JvA	GACCGTTATAGTTATTTAAATTGTTTAACTTGG
AR152	ACACTCTTTCCCTACACGACGCTCTTCCGATCT
168_JvA	/5′phos/NNNNNNNNNNNNNNNNNNNNGATCATGCTAGTTGTGGTTTGTC
169_JvA	AGATCGGAAGAGCGTCGTGTAGGGAAAGAGTGTCAGTGAAAAAAATGCTT
151AR	AATGATACGGCGACCACCGAGATCTACACTCTTTCCCTACACGACGCTCTTCCGATCT
219AR	GTGACTGGAGTTCAGACGTGTGCTCTTCCGATCTCTAAGGTGTATGTAAACTTCCGACTTCAACTG
iPCR indexing	CAAGCAGAAGACGGCATACGAGATNNNNNNGTGACTGGAGTTCAGACGTGTGCTCTTCCGATC
Library indexing	CAAGCAGAAGACGGCATACGAGATNNNNNNGTGACTGGAGTTCAGACGTGTGCTCTTCCGATCTGCACGCCTTCAAGACCCCCATCGCC
DamID adapter top	CTAATACGACTCACTATAGGGCAGCGTGGTCGCGGCCGAGGA
DamID adapter bottom	TCCTCGGCCGCG
Adr-PCR-Rand1	NNNNGTGGTCGCGGCCGAGGATC
Y-adaptor top	ACACTCTTTCCCTACACGACGCTCTTCCGATCT
Y-adaptor bottom	/5′phos/GATCGGAAGAGCACACGTCT
Illumina index	CAAGCAGAAGACGGCATACGAGNNNNNNNNGTGACTGGAGTTCAGACGTGTGCTCTTCCGATCT
P5-Illumina-2	AATGATACGGCGACCACCGAGATCTACACTCTTTCCCTACACGACGCTCTTCCGATCT
45JvA	ATTCTGATATTTCACATTCTTAAAATAAAGTGG
94JvA	ACCGTTATAGTTATTAACTTGGGTCAAACATTT

### qPCR

qPCR was performed using SensiFAST SYBR No-ROX Kit (Bioline) with 300 nM forward and reverse primer in 10 µl volume. Detection was performed in a LightCycler 480 (Roche) under the following qPCR conditions: 95 °C for 2 min (1×), 95 °C for 5 s, 60 °C for 10 s, 72 °C for 10 s (45×). Ct values were normalized to a housekeeping gene (*tsr*).

### Spike-in for expression normalization

An independent TRIP pool with a complexity of approximately 200 barcodes was suspended in TRIsure (Bioline) and added to TRIP cells collected for cDNA extraction to an amount of 2.5 % based on cell counts.

### Library preparation for next-generation sequencing

For cDNA reads, RNA from 10 × 10^6^ transfected cells isolated by FACS sorting was extracted and transcribed into cDNA. One-fourth of total cDNA was used in a PCR to add sequencing adapters. Before sequencing library preparation, the appropriate PCR cycle number was determined by qPCR to avoid overamplification. For gDNA reads, gDNA was isolated from 10 × 10^6^ unsorted TRIP cells using ISOLATE II Genomic DNA Kit (Bioline). 500 ng was used in a PCR to add sequencing adapters and amplified over 25 cycles. PCR was performed using Phusion polymerase (2 U, Thermo Scientific) in GC buffer with 500 nM forward and reverse primer (library indexing primers and 151AR) and dNTPs (200 µM each) in a total volume of 100 µl. PCR conditions were 1 min at 98 °C (1×), followed by 15 s at 98 °C, 30 s at 68 °C and 30 s at 72 °C (cycle number as determined by qPCR) followed by 3 min at 72 °C. PCR products were purified using the ISOLATE II PCR and Gel Kit (Bioline).

### Next-generation sequencing

Before next-generation sequencing, product size selection was performed using 2 % E-Gel SizeSelect Gels (Thermo Scientific). For expression and normalization reads, 6 cDNA samples and 6 gDNA samples were multiplexed in one lane and sequenced with 65-bp single reads using a HiSeq2500 (Illumina) yielding 210 × 10^6^ reads. For integration mapping, triplicate indexed PCR samples were pooled and sequenced with 75-bp paired-end reads using a MiSeq (Illumina) yielding 24 × 10^6^ reads. Reads were mapped to *Drosophila melanogaster* genome release dm3 using Bowtie.

### Data analysis

Extraction of 21-nt barcode reads from fastq files and alignment of TRIP mapping reads was performed using the TRIP script available at http://trip.nki.nl. We set a Hamming distance of 2 for removing mutated barcodes, a maximum distance of 500 nt on the forward read and 20 nt on the reverse read to cluster the positions during mapping and a minimum read number of 1. We obtained a total of 27/28 × 10^6^ barcoded cDNA reads and 41/46 × 10^6^ barcoded gDNA reads for two replicate experiments of day 2 after transfection. Spike-in library gDNA was sequenced separately to determine the spike-in barcode sequences. A list of the most abundant spike-in barcodes with over 1000 counts in gDNA was used for extracting spike-in reads from the TRIP samples. cDNA read counts per barcode were divided by the sum of cDNA read counts for all spike-in barcodes in that sample to correct for sequencing depth and standardize expression between samples. gDNA read counts were transformed to counts per million (cpm) to correct for sequencing depth. Spike-in corrected cDNA counts were then divided by gDNA cpm counts which yields the normalized expression per barcode. We set a cutoff for barcodes that had at least 100 gDNA reads in all samples to ensure that each barcode was sufficiently represented in the pool. After cutoff for gDNA counts, we worked with an average of 1200/1500 cDNA reads per barcode in replicate 1/2 of the Gal4-HP1-transfected samples and 920/1400 and 1700/1900 for the Gal4- and HP1-transfected control samples. For gDNA counts, we obtained an average of 1900/2800 reads per barcode of the Gal4-HP1-transfected samples and 2900/2800 and 2200/2500 for the Gal4- and HP1-transfected controls. To ensure reliable allocation of barcode integrations, we worked with a total of 1093 barcodes that fit the following criteria: (1) more than 2 reads in both forward and reverse mapping, (2) 80 % of forward and reverse mapping reads matched with the first mapping location and less than 10 % of forward and reverse mapping reads matched with the second mapping location and (3) mapq score of 10 or higher for forward and reverse mapping. Sleeping Beauty is known not to generate tandem integrations; moreover, by exclusion of integrations with non-concordant forward and reverse mapping reads we exclude potential tandem integration events. As we observed good correlation between replicate experiments, we performed all further analysis on the mean normalized expression per barcode. For barcodes with 0 reads in one replicate, we set the mean normalized expression to 0. Data analysis was done in RStudio, R version 3.2.1.

### Immunofluorescence microscopy

Kc167 cells were collected 2 days after transfection, resuspended in serum-free medium and allowed to settle on coverslips coated with poly-d-lysine (Sigma-Aldrich) for 1 h. Cells were washed with PBS and fixed with 2 % paraformaldehyde (Sigma-Aldrich) for 10 min. After washing with PBS, cells were permeabilized with 0.5 % NP-40 in PBS for 10 min and washed with PBS, and coverslips were transferred to a wet chamber. Coverslips were blocked with PBG (0.5 % of 0.2 % cold water fish gelatin with 0.2 % sodium azide in PBS) for 15 min and then incubated with anti-HP1a primary antibody at 386 ng/ml concentration (C1A9, Developmental Studies Hybridoma Bank of the University of Iowa) for 1 h. After four 5-min washes with PBG, coverslips were incubated with 1:200-diluted DyLight 594-coupled secondary antibody (715-515-150, Jackson ImmunoResearch) for 1 h. After washing with PBG and PBS, coverslips were mounted to objective slides in Vectashield mounting medium with DAPI (Vector Laboratories). Images were taken using an Axio Observer (Zeiss) with a 40× objective and oil immersion and processed with Zen software.

### Western blot

Cells transfected with Gal4-HP1, HP1 or Gal4 plasmids were isolated by FACS sorting by 2, 4 and 6 days after transfection. Cells were kept on ice and lysed for protein extraction in lysis buffer (10 mM KCl, 1.5 mM MgCl2, 10 mM Tris, 10 % SDS) supplemented with proteinase inhibitor (cOmplete, Roche). 40 µg per sample was diluted in Laemmli buffer (50 mM Tris–HCl pH 6.8, 2 % SDS, 10 % glycerol, 1 % beta-mercaptoethanol, 12.5 mM EDTA, 0.02 % bromophenol blue), denatured at 90 °C for 5 min, separated on a 16 % SDS-PAGE gel and blotted on nitrocellulose membrane (Amersham Protran 0.45 µM). HP1a was detected using monoclonal antibody C1A9 obtained from the Developmental Studies Hybridoma Bank of the University of Iowa at 386 ng/ml concentration. H3 was detected using antibody ab1791 obtained from Abcam at 33 ng/ml concentration. Quantitative western blot analysis was performed using the LI‐COR Odyssey IRDye^®^ IR imager (Biosciences), IRDye secondary antibodies and the Odyssey LI‐COR software.

### MboI assay for reporter accessibility

1 × 10^6^ cells of each single-integration cell line with GFP reporter integrations were transfected with Gal4-Dam and incubated for 2 days. gDNA was extracted using ISOLATE II Genomic DNA Kit (Bioline) according to manufacturer’s instruction, and 500 ng was digested with 10 units of each MboI and NaeI for 1 h at 37 °C in CutSmart buffer (New England Biolabs) in a total volume of 25 µl. 20 ng digested DNA was used in a qPCR with primers “upstream,” “GFP” and “TSR.” Amplicons of “upstream” and “GFP” primers each cover one GATC site and can therefore be used to detect DAM methylation levels, whereas the amplicon of primers “TSR” does not contain GATC and was therefore used for normalization. Methylation levels were calculated as percentage of averages of negative and positive controls.

### DamID-seq

20 × 10^6^ wild-type Kc167 cells were transfected in duplicates with 20 µg plasmids expressing Dam-HP1 or Dam only and collected 2 days after transfection. gDNA from 5 × 10^6^ cells was isolated using ISOLATE II Genomic DNA Kit (Bioline) according to manufacturer’s instruction. 500 ng gDNA was digested with DpnI (10 U, New England Biolabs) in CutSmart buffer in a total volume of 20 µl at 37 °C for 8 h. Reaction was terminated by heat inactivation at 80 °C for 20 min. Fragments were ligated to 12.5 pmol DamID adapters using T4 ligase (2.5 U, New England Biolabs) in T4 ligase buffer in a total volume of 25 µl incubated at 16 °C for 16 h. The reaction was heat inactivated for 10 min at 65 °C. Products were then digested with DpnII to remove partially methylated fragments. DpnII buffer and DpnII (10 U, New England Biolabs) were added in a total volume of 80 µl and incubated at 37 °C for 1 h. 20 µl of DpnII-digested products was amplified by PCR with MyTaq Red Mix (Bioline) and 2.5 µM primers Adr-PCR-Rand1 in a total volume of 80 µl. PCR settings were 8 min at 72 °C (1×) followed by 20 s at 94 °C, 30 s at 58 °C, 20 s at 72 °C (15×) and 2 min at 72 °C (1×). PCR products were cleaned up using the ISOLATE II PCR and Gel Kit (Bioline) according to the manufacturer’s instructions and eluted in 26 µl H_2_O. Fragment ends were blunted using the End-It™ DNA End-Repair Kit (Epicentre) in a 50-µl reaction according to the manufacturer’s instructions. Products were cleaned up using the ISOLATE II PCR and Gel Kit (Bioline) and eluted in 26 µl H_2_O. For adding a 3′ overhang, fragments were treated with Klenow fragment (3′-5′ exo-, 25 U, New England Biolabs) in buffer 2 with 200 µM dATP (Thermo Fisher Scientific) in a 50-µl reaction incubated at 37 °C for 30 min. Reaction was terminated by heat inactivation at 75 °C for 20 min. Products were purified using CleanPCR magnetic beads (CleanNA) according to the manufacturer’s instructions and eluted in 20 µl H_2_O. 260 ng fragments were ligated to 2.5 µM Y-adaptors using T4 ligase (2.5 U, New England Biolabs) in T4 ligase buffer in a total volume of 10 µl incubated at 16 °C for 16 h. Reaction was terminated by heat inactivation at 65 °C for 10 min. Products were purified using CleanPCR magnetic beads (CleanNA) according to the manufacturer’s instructions and eluted in 20 µl H_2_O. Adapters and indices for next-generation sequencing and multiplexing were added by PCR using 8 µl of purified fragments in MyTaq Red mix together with 250 nM Illumina index primers and 250 nM primer P5-Illumina-2 in a total volume of 20 µl. PCR settings were 1 min at 94 °C (1×) followed by 30 s at 94 °C, 30 s at 58 °C, 30 s at 72 °C (11×) and 2 min at 72 °C (1×). PCR products were pooled at equal amounts, purified using CleanPCR magnetic beads (CleanNA) and eluted in 20 µl H_2_O. Pooled samples of two replicates transfected with Dam-HP1 or Dam only were sequenced with 69 × 10^6^ reads in total.

Reads were filtered for sequences containing DamID adapter sequence using cutadapt, aligned to *Drosophila* genome release dm3 using Bowtie 2 and matched with GATC-flanked fragments which yielded 6–11 × 10^6^ fragments per sample. As replicate fragment counts were highly correlated (*r*_*p*_ = 0.99), we worked with mean counts of two Dam-HP1 replicates normalized to mean of Dam only replicates for further analysis.

### Inducible expression experiment with single-integration cell line

GFP reporter expression in single-integration cell line “C” was induced by adding CuSO_4_ to a final concentration of 0.005, 0.05 and 0.5 mM. The experiment was performed in three replicates. Two days after induction, 20 × 10^6^ cells were transfected with plasmids encoding Gal4-HP1 or Gal4 only. Two days after transfection, transfected cells were isolated by FACS sorting for mCherry. GFP expression was measured by qPCR using primers “GFP” and normalized to housekeeping gene *tsr* using primers “TSR.”

## Additional files

**Additional file 1: Figure S1.** Correlation of normalized expression between replicate experiments.

**Additional file 2: Figure S2.** Expression of integrated reporters as quantified by NGS in the Gal4-transfected control condition divided over five chromatin states. Median values are represented by black horizontal bars. Number of reporters integrated in each state is specified above graph.

**Additional file 3: Figure S3.** GFP expression and transfection rate (measured via mCherry) of TRIP pool as quantified by FACS by days 2, 4 and 6 after transfection.

**Additional file 4: Figure S4.** Western blot of Kc167 cells transiently transfected with Gal4-V5-HP1 and V5-HP1 or untransfected control (UT) stained with anti-HP1a and anti-H3.

**Additional file 5: Figure S5.** Chromosome ideograms of TRIP reporter integration sites. Colors represent chromatin state at the integration site according to the nine-state model. Centromere position is indicated by black triangle. Scatter plot above ideogram shows fold downregulation upon Gal4-HP1 tethering.

**Additional file 6: Figure S6.** Fold change in reporter expression Gal4-HP1/HP1 as quantified by NGS divided over nine chromatin states. Median values are represented by black horizontal bars. Number of reporters integrated in each state is specified above graph.

**Additional file 7: Figure S7.** Localization of transiently transfected constructs. Gal4-V5-HP1, Gal4-V5 and V5-HP1 in Kc167 cells as detected by immunofluorescence with anti-V5 (green) and DAPI (blue). Transfected cells express mCherry (red).

## Abbreviations

FACS: fluorescence-activated cell sorting
Gal4DBD: Gal4 DNA-binding domain
gDNA: genomic DNA
GFP: green fluorescent protein
HP1: heterochromatin protein 1
NGS: next-generation sequencing
pMT: metallothionein promoter
TRIP: thousands of reporters integrated in parallel

## References

[1] Filion GJ, van Bemmel JG, Braunschweig U, Talhout W, Kind J, Ward LD, Brugman W, de Castro IJ, Kerkhoven RM, Bussemaker HJ, van Steensel B (2010). Systematic protein location mapping reveals five principal chromatin types in *Drosophila* cells. Cell.

[2] Kharchenko PV, Alekseyenko AA, Schwartz YB, Minoda A, Riddle NC, Ernst J, Sabo PJ, Larschan E, Gorchakov AA, Gu T, Linder-Basso D, Plachetka A, Shanower G, Tolstorukov MY, Luquette LJ, Xi R, Jung YL, Park RW, Bishop EP, Canfield TK, Sandstrom R, Thurman RE, MacAlpine DM, Stamatoyannopoulos JA, Kellis M, Elgin SCR, Kuroda MI, Pirrotta V, Karpen GH, Park PJ (2011). Comprehensive analysis of the chromatin landscape in *Drosophila melanogaster*. Nature.

[3] Eissenberg JC, James TC, Foster-Hartnett DM, Hartnett T, Ngan V, Elgin SC (1990). Mutation in a heterochromatin-specific chromosomal protein is associated with suppression of position-effect variegation in *Drosophila melanogaster*. Proc Natl Acad Sci USA.

[4] Verschure PJ, van der Kraan I, de Leeuw W, van der Vlag J, Carpenter AE, Belmont AS, van Driel R (2005). In vivo HP1 targeting causes large-scale chromatin condensation and enhanced histone lysine methylation. Mol Cell Biol.

[5] Canzio D, Chang EY, Shankar S, Kuchenbecker KM, Simon MD, Madhani HD, Narlikar GJ, Al-Sady B (2011). Chromodomain-mediated oligomerization of HP1 suggests a nucleosome-bridging mechanism for heterochromatin assembly. Mol Cell.

[6] Yin H, Sweeney S, Raha D, Snyder M, Lin H (2011). A high-resolution whole-genome map of key chromatin modifications in the adult *Drosophila melanogaster*. PLoS Genet.

[7] de Wit E, Greil F, van Steensel B (2005). Genome-wide HP1 binding in *Drosophila*: developmental plasticity and genomic targeting signals. Genome Res.

[8] Yasuhara JC, Wakimoto BT (2006). Oxymoron no more: the expanding world of heterochromatic genes. Trends Genet.

[9] Kwon SH, Workman JL (2011). The changing faces of HP1: from heterochromatin formation and gene silencing to euchromatic gene expression: HP1 acts as a positive regulator of transcription. BioEssays.

[10] Schotta G, Ebert A, Dorn R, Reuter G (2003). Position-effect variegation and the genetic dissection of chromatin regulation in *Drosophila*. Semin Cell Dev Biol.

[11] Fischle W, Tseng BS, Dormann HL, Ueberheide BM, Garcia BA, Shabanowitz J, Hunt DF, Funabiki H, Allis CD (2005). Regulation of HP1-chromatin binding by histone H3 methylation and phosphorylation. Nature.

[12] Wang C, Cai W, Li Y, Deng H, Bao X, Girton J, Johansen J, Johansen KM (2011). The epigenetic H3S10 phosphorylation mark is required for counteracting heterochromatic spreading and gene silencing in *Drosophila melanogaster*. J Cell Sci.

[13] Akhtar W, de Jong J, Pindyurin AV, Pagie L, Meuleman W, de Ridder J, Berns A, Wessels LFA, van Lohuizen M, van Steensel B (2013). Chromatin position effects assayed by thousands of reporters integrated in parallel. Cell.

[14] Pan T, Coleman JE (1989). Structure and function of the Zn(II) binding site within the DNA-binding domain of the GAL4 transcription factor. Proc Natl Acad Sci USA.

[15] Sadowski I, Ma J, Triezenberg S, Ptashne M (1988). GAL4-VP16 is an unusually potent transcriptional activator. Nature.

[16] Margolin JF, Friedman JR, Meyer WK, Vissing H, Thiesen HJ, Rauscher FJ (1994). Krüppel-associated boxes are potent transcriptional repression domains. Proc Natl Acad Sci USA.

[17] Li Y, Danzer JR, Alvarez P, Belmont AS, Wallrath LL (2003). Effects of tethering HP1 to euchromatic regions of the *Drosophila* genome. Development.

[18] Dialynas G, Speese S, Budnik V, Geyer PK, Wallrath LL (2010). The role of *Drosophila* Lamin C in muscle function and gene expression. Development.

[19] Danzer JR, Wallrath LL (2004). Mechanisms of HP1-mediated gene silencing in *Drosophila*. Development.

[20] Seum C, Delattre M, Spierer A, Spierer P (2001). Ectopic HP1 promotes chromosome loops and variegated silencing in *Drosophila*. EMBO J.

[21] van Steensel B, Henikoff S (2000). Identification of in vivo DNA targets of chromatin proteins using tethered dam methyltransferase. Nat Biotechnol.

[22] Zhang W, Deng H, Bao X, Lerach S, Girton J, Johansen J, Johansen KM (2006). The JIL-1 histone H3S10 kinase regulates dimethyl H3K9 modifications and heterochromatic spreading in *Drosophila*. Development.

[23] Elgin SCR, Reuter G (2013). Position-effect variegation, heterochromatin formation, and gene silencing in *Drosophila*. Cold Spring Harb Perspect Biol.

[24] Hathaway NA, Bell O, Hodges C, Miller EL, Neel DS, Crabtree GR (2012). Dynamics and memory of heterochromatin in living cells. Cell.

[25] Ayyanathan K, Lechner MS, Bell P, Maul GG, Schultz DC, Yamada Y, Tanaka K, Torigoe K, Rauscher FJ (2003). Regulated recruitment of HP1 to a euchromatic gene induces mitotically heritable, epigenetic gene silencing: a mammalian cell culture model of gene variegation. Genes Dev.

[26] Ritchie ME, Phipson B, Wu D, Hu Y, Law CW, Shi W, Smyth GK: Limma powers differential expression analyses for RNA-sequencing and microarray studies. Nucleic Acids Res 2015:gkv007. 10.1093/nar/gkv007 PMC4402510 25605792

[27] Piacentini L, Fanti L, Berloco M, Perrini B, Pimpinelli S (2003). Heterochromatin protein 1 (HP1) is associated with induced gene expression in *Drosophila euchromatin*. J Cell Biol.

[28] Cryderman DE, Grade SK, Li Y, Fanti L, Pimpinelli S, Wallrath LL (2005). Role of *Drosophila* HP1 in euchromatic gene expression. Dev Dyn.

[29] Lundberg LE, Stenberg P, Larsson J (2013). HP1a, Su(var)3–9, SETDB1 and POF stimulate or repress gene expression depending on genomic position, gene length and expression pattern in *Drosophila melanogaster*. Nucleic Acids Res.

[30] Yoh SM, Lucas JS, Jones KA (2008). The Iws1:Spt6:CTD complex controls cotranscriptional mRNA biosynthesis and HYPB/Setd2-mediated histone H3K36 methylation. Genes Dev.

[31] Sun X-J, Wei J, Wu X-Y, Hu M, Wang L, Wang H-H, Zhang Q-H, Chen S-J, Huang Q-H, Chen Z (2005). Identification and characterization of a novel human histone H3 lysine 36-specific methyltransferase. J Biol Chem.

[32] Pan L, Xie W, Li K-L, Yang Z, Xu J, Zhang W, Liu L-P, Ren X, He Z, Wu J, Sun J, Wei H-M, Wang D, Xie W, Li W, Ni J-Q, Sun F-L (2015). Heterochromatin remodeling by CDK12 contributes to learning in *Drosophila*. Proc Natl Acad Sci USA.

[33] Nakayama J, Klar AJ, Grewal SI (2000). A chromodomain protein, Swi6, performs imprinting functions in fission yeast during mitosis and meiosis. Cell.

[34] Hall IM, Shankaranarayana GD, Noma K-I, Ayoub N, Cohen A, Grewal SIS, Reik W, Walter J, Grewal SIS, Elgin SC, Cohen DE, Lee JT, Cavalli G, Henikoff S, Strahl BD, Allis CD, Jenuwein T, Allis CD, Birchler JA, Bhadra MP, Bhadra U, Hsieh J, Fire A, Dorer DR, Henikoff S, Chikashige Y, Hahnenberger KM, Carbon J, Clarke L, Allshire RC (2002). Establishment and maintenance of a heterochromatin domain. Science.

[35] Audergon PNCB, Catania S, Kagansky A, Tong P, Shukla M, Pidoux AL, Allshire RC (2015). Restricted epigenetic inheritance of H3K9 methylation. Science (80-).

[36] Ragunathan K, Jih G, Moazed D (2014). Epigenetic inheritance uncoupled from sequence-specific recruitment. Science (80-).

[37] de Jong J, Akhtar W, Badhai J, Rust AG, Rad R, Hilkens J, Berns A, van Lohuizen M, Wessels LFA, de Ridder J (2014). Chromatin landscapes of retroviral and transposon integration profiles. PLoS Genet.

[38] Greil F, van der Kraan I, Delrow J, Smothers JF, de Wit E, Bussemaker HJ, van Driel R, Henikoff S, van Steensel B (2003). Distinct HP1 and Su(var)3–9 complexes bind to sets of developmentally coexpressed genes depending on chromosomal location. Genes Dev.

[39] Tolhuis B, de Wit E, Muijrers I, Teunissen H, Talhout W, van Steensel B, van Lohuizen M (2006). Genome-wide profiling of PRC1 and PRC2 Polycomb chromatin binding in *Drosophila melanogaster*. Nat Genet.

[40] van Bemmel JG, Filion GJ, Rosado A, Talhout W, de Haas M, van Welsem T, van Leeuwen F, van Steensel B (2013). A network model of the molecular organization of chromatin in *Drosophila*. Mol Cell.

[41] González M, Martín-Ruíz I, Jiménez S, Pirone L, Barrio R, Sutherland JD (2011). Generation of stable *Drosophila* cell lines using multicistronic vectors. Sci Rep.

[42] Kind J, Pagie L, Ortabozkoyun H, Boyle S, de Vries SS, Janssen H, Amendola M, Nolen LD, Bickmore WA, van Steensel B (2013). Single-cell dynamics of genome–nuclear lamina interactions. Cell.

[43] Bateman JR, Lee AM, Wu C (2006). Site-specific transformation of *Drosophila* via phiC31 integrase-mediated cassette exchange. Genetics.

