# Supplementary figures and images for: High-throughput assessment of context-dependent effects of chromatin proteins
Source: Epigenetics Chromatin. 2016 Oct 18;9:43. doi: 10.1186/s13072-016-0096-y (PMC5069885; doi:10.1186/s13072-016-0096-y)

**S1****Gal4HP1**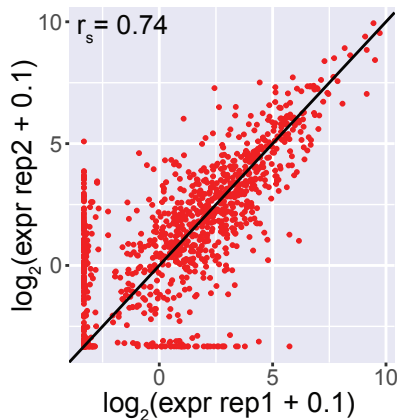**Gal4**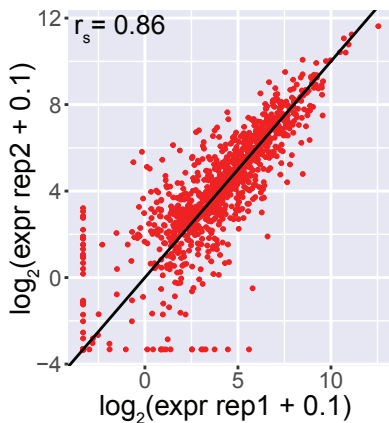**HP1**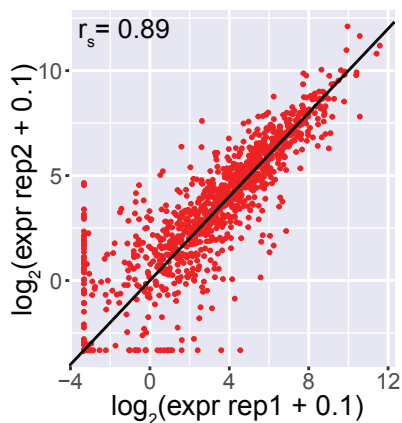

Supplement: Supplementary file 1 — Additional file 1: Figure S1. Correlation of normalized expression between replicate experiments. [file 13072_2016_96_MOESM1_ESM.pdf]

# S2

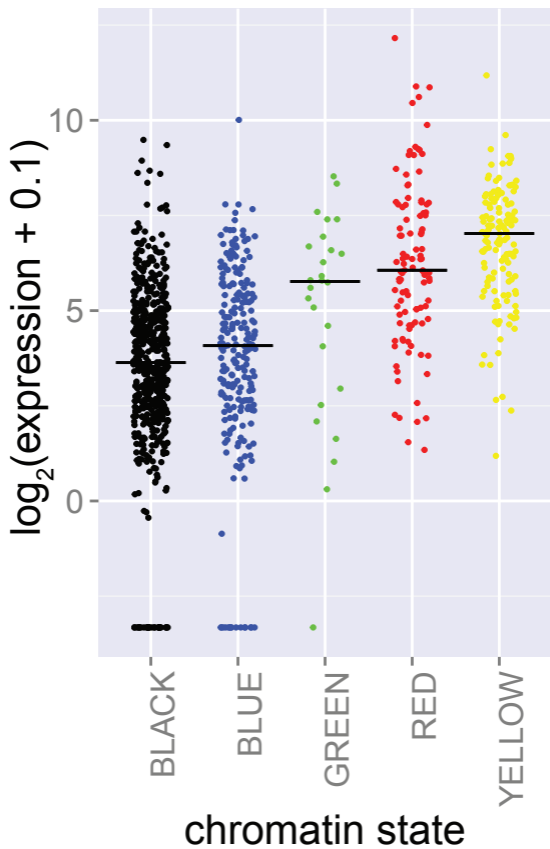

Supplement: Supplementary file 2 — Additional file 2: Figure S2. Expression of integrated reporters as quantified by NGS in the Gal4-transfected control condition divided over five chromatin states. Median values are represented by black horizontal bars. Number of reporters integrated in each state is specified above graph. [file 13072_2016_96_MOESM2_ESM.pdf]

# S3

## Gal4-HP1

## Gal4

## HP1

Day 2

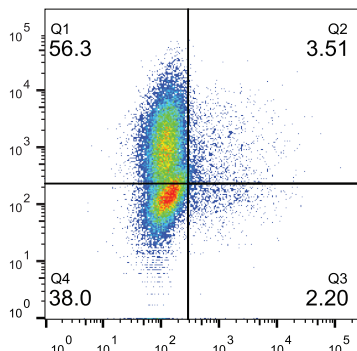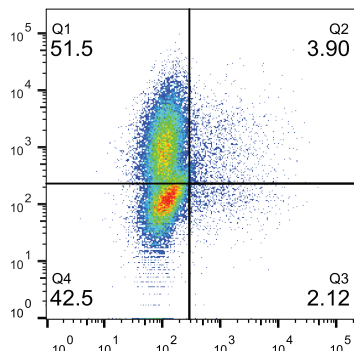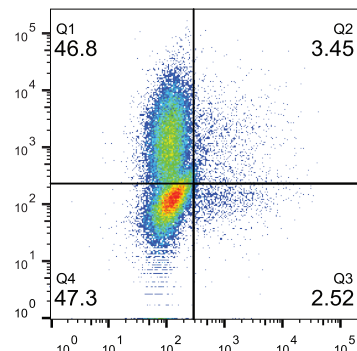

Day 4

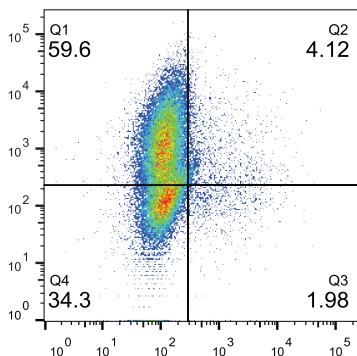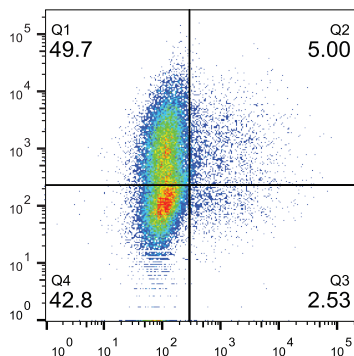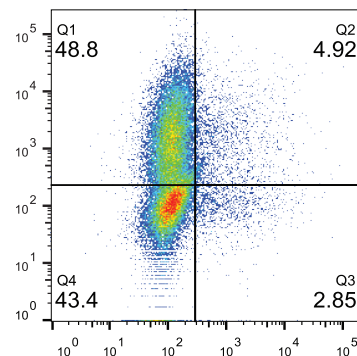

Day 6

↑ mCherry

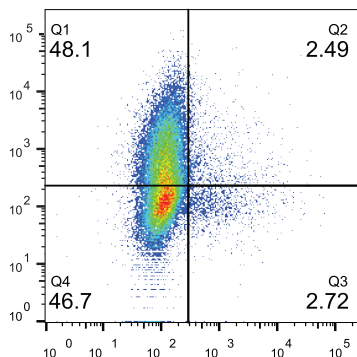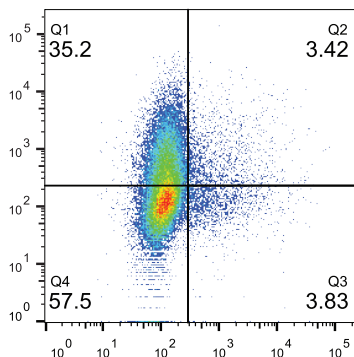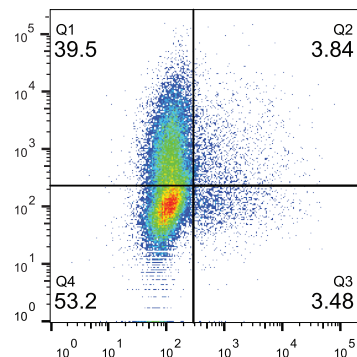

→ GFP

Supplement: Supplementary file 3 — Additional file 3: Figure S3. GFP expression and transfection rate (measured via mCherry) of TRIP pool as quantified by FACS by days 2, 4 and 6 after transfection. [file 13072_2016_96_MOESM3_ESM.pdf]

# S4

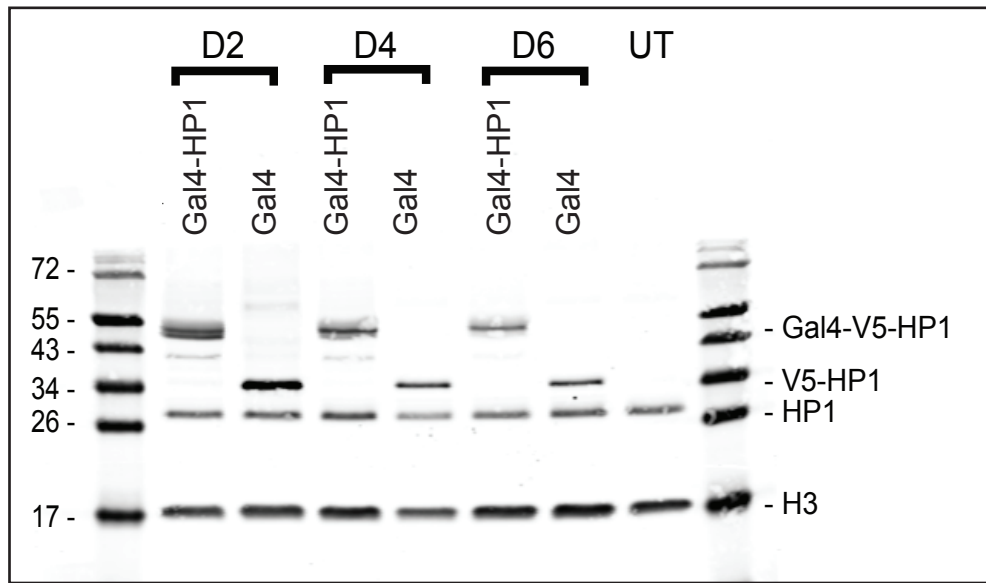

Supplement: Supplementary file 4 — Additional file 4: Figure S4. Western blot of Kc167 cells transiently transfected with Gal4-V5-HP1 and V5-HP1 or untransfected control (UT) stained with anti-HP1a and anti-H3. [file 13072_2016_96_MOESM4_ESM.pdf]

# S5

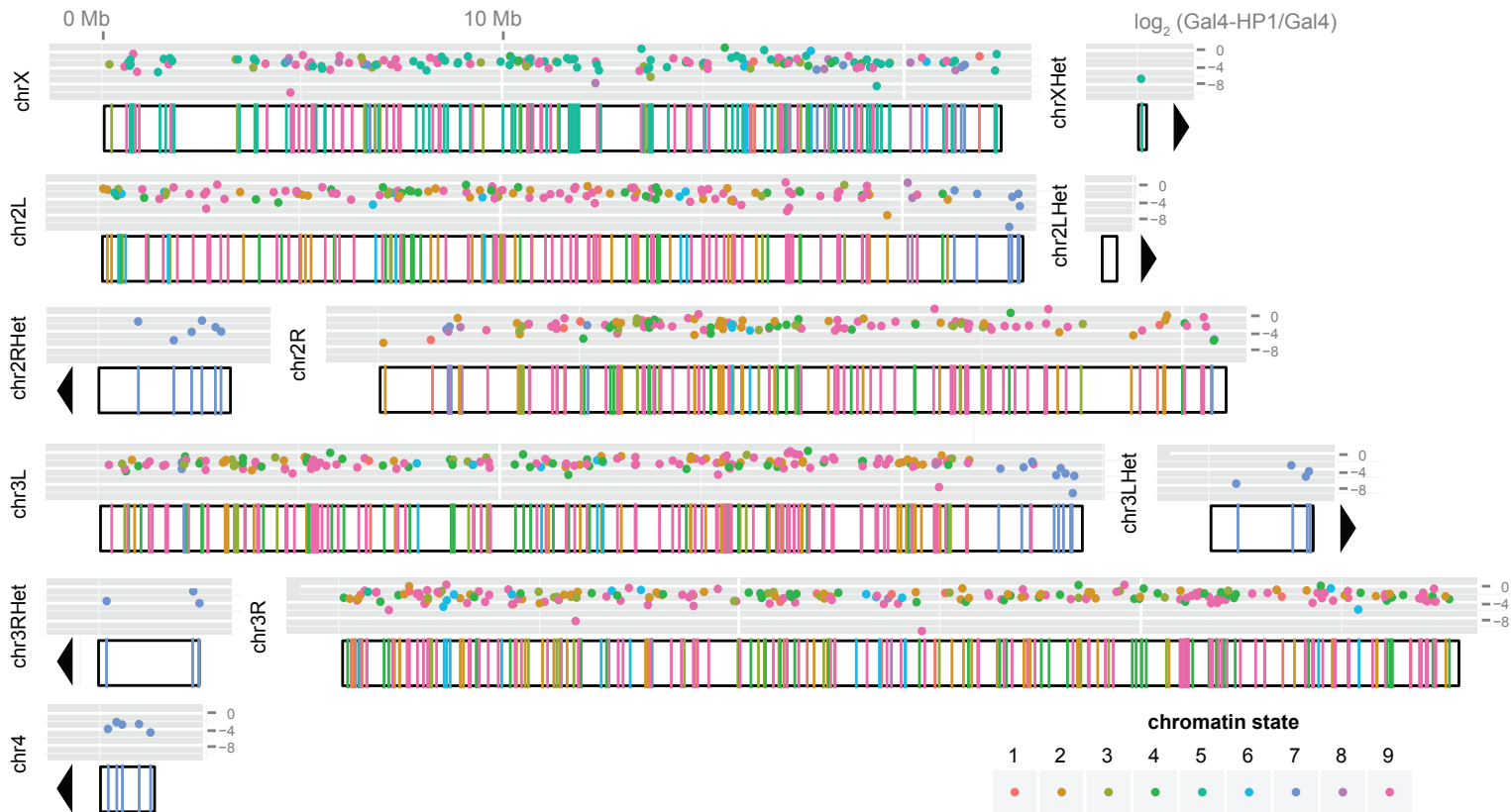

Supplement: Supplementary file 5 — Additional file 5: Figure S5. Chromosome ideograms of TRIP reporter integration sites. Colors represent chromatin state at the integration site according to the nine-state model. Centromere position is indicated by black triangle. Scatter plot above ideogram shows fold downregulation upon Gal4-HP1 tethering. [file 13072_2016_96_MOESM5_ESM.pdf]

**S6**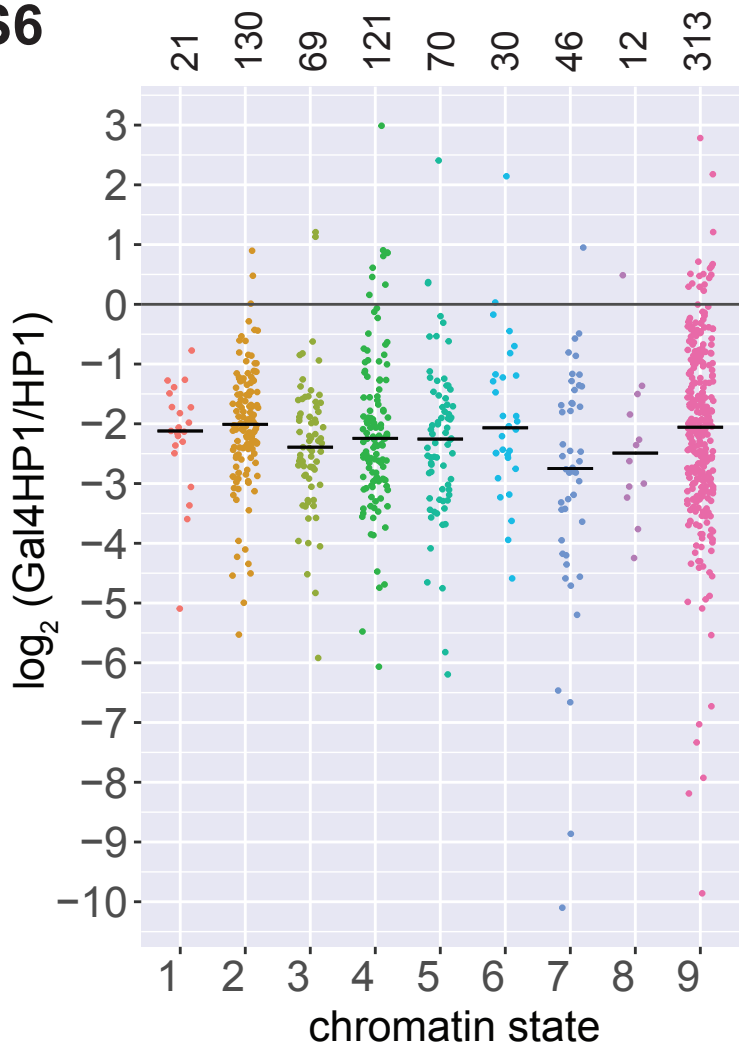

Supplement: Supplementary file 6 — Additional file 6: Figure S6. Fold change in reporter expression Gal4-HP1/HP1 as quantified by NGS divided over nine chromatin states. Median values are represented by black horizontal bars. Number of reporters integrated in each state is specified above graph. [file 13072_2016_96_MOESM6_ESM.pdf]

# S7

## Gal4-HP1

## Gal4

## HP1

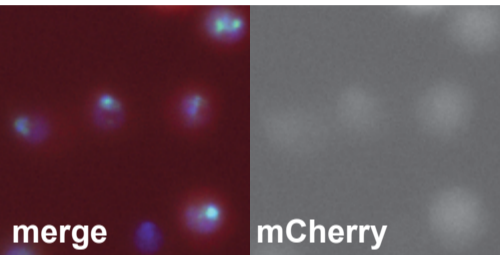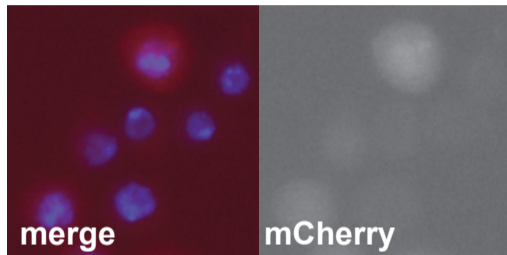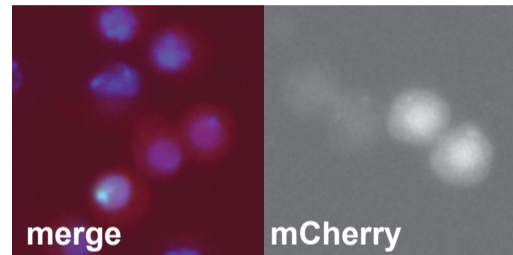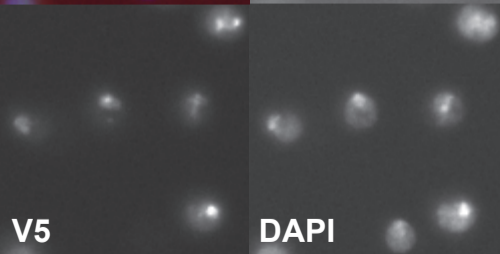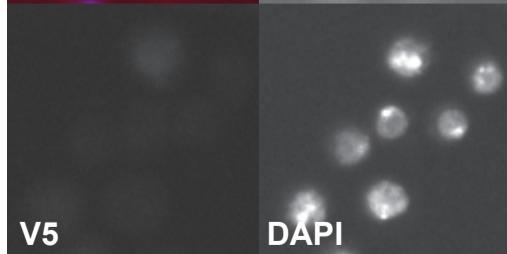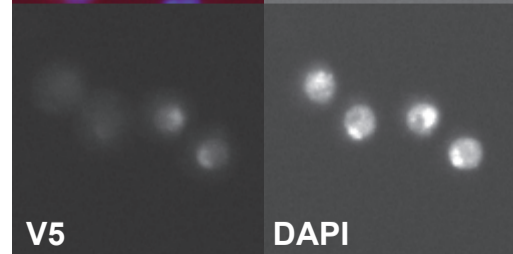

Supplement: Supplementary file 7 — Additional file 7: Figure S7. Localization of transiently transfected constructs. Gal4-V5-HP1, Gal4-V5 and V5-HP1 in Kc167 cells as detected by immunofluorescence with anti-V5 (green) and DAPI (blue). Transfected cells express mCherry (red). [file 13072_2016_96_MOESM7_ESM.pdf]
